# Integrated bioinformatics and network pharmacology to explore the therapeutic target and molecular mechanisms of Bailing capsule on polycystic ovary syndrome

**DOI:** 10.1186/s12906-023-04280-6

**Published:** 2023-12-15

**Authors:** Hao-ru Guan, Bo Li, Ze-hua Zhang, Han-song Wu, Xing-lishang He, Ying-jie Dong, Jie Su, Gui-yuan Lv, Su-hong Chen

**Affiliations:** 1 Collaborative Innovation Center of Yangtze River Delta Region Green Pharmaceuticals, Zhejiang University of Technology, Hangzhou, Zhejiang 310014 PR China; 2 College of Pharmaceutical Science, Zhejiang Chinese Medical University, Hangzhou, Zhejiang 310053 PR China; 3 Zhejiang Provincial Key Laboratory of TCM for Innovative R & D and Digital Intelligent Manufacturing of TCM Great Health Products, Huzhou, Zhejiang Province 313200 PR China

**Keywords:** Polycystic ovary syndrome, Bailing capsule, Bioinformatics, Microarray, Network pharmacology, Gene expression

## Abstract

**Background:**

Polycystic ovary syndrome (PCOS) is a complex endocrine and metabolic disorder that is common in women of reproductive age. The clinical features of PCOS include hyperandrogenemia and polycystic ovarian changes. Bailing capsule (BL), a proprietary Chinese medicine that contains fermented *Cordyceps sinensis* powder, has been applied to treat PCOS. However, the specific active ingredients of BL and its mechanisms of action are yet to be elucidated.

**Methods:**

Initially, the effectiveness of BL on PCOS model mice was evaluated. Subsequently, the active ingredients of BL were searched in the TCMSP and TCM Systems Pharmacology databases, and their targets were predicted using Swiss Target Prediction and SEA databases. Furthermore, the GEO gene database was used to screen for differentially expressed genes (DEGs) related to PCOS. Data from Gene Card, OMIM, DDT, and Drugbank databases were then combined to establish a PCOS disease gene library. Cross targets were imported into the STRING database to construct a protein–protein interaction network. In addition, GO and KEGG pathway enrichment analyses were performed using Metascape and DAVID databases and visualized using Cytoscape software and R 4.2.3. The core targets were docked with SYBYL-X software, and their expressions in PCOS mice were further verified using qPCR.

**Results:**

The core active ingredients of BL were identified to be linoleyl acetate, cholesteryl palmitate, arachidonic acid, among others. Microarray data sets from four groups containing disease and normal samples were obtained from the GEO database. A total of 491 DEGs and 106 drug–disease cross genes were selected. Estrous cycle and ovarian lesions were found to be improved in PCOS model mice following BL treatment. While the levels of testosterone, progesterone, and prolactin decreased, that of estradiol increased. qPCR findings indicated that the expressions of *JAK2*, *PPARG*, *PI3K*, and *AKT1* were upregulated, whereas those of *ESR1* and *IRS1* were downregulated in PCOS model mice. After the administration of BL, the expressions of associated genes were regulated. This study demonstrated that BL exerted anti-PCOS effects via PIK3CA, ESR1, AKT, PPARG, and IRS1 targets affecting PI3K-Akt signaling pathways.

**Discussion:**

This research clarified the multicomponent, multitarget, and multichannel action of BL and provided a theoretical reference for further investigations on its pharmacological basis and molecular mechanisms against PCOS.

## Background

Polycystic ovary syndrome (PCOS) is a heterogeneous endocrinological and metabolic disorder that occurs commonly in women of reproductive age. The main clinical symptoms include abnormal ovulation, ovarian changes, and androgen increase [1, 2]. The development of PCOS is often accompanied by complications pertaining to infertility, insulin resistance (IR), abdominal fat accumulation, nonalcoholic fatty liver disease, obesity, and cardiovascular disease [3–5]. The incidence of PCOS in women is > 20%, and according to the most recent diagnostic criterion, it is now the most prevalent endocrine and metabolic condition in women of reproductive age [6].

The pathogenesis and causes of the disease have not been completely examined and may be linked to genetic and environmental factors. The drugs used to treat PCOS are mostly symptomatic interventions, such as oral contraceptives, antiandrogens, and insulin sensitizers [7]. This treatment strategy is flexible in terms of dosing, can be adapted to different symptoms, and is supported by a large body of clinical evidence, which ensures drug safety [8–10].

In recent years, the efficacy of several classical Chinese medicine prescriptions in treating PCOS has been increasingly recognized [11–14]. According to a study, the pregnancy rate for patients with PCOS treated using herbal medications was 37.14% and the rate of pregnant women giving birth to healthy babies was 34.97%. Moreover, herbal medications can alleviate endocrine and metabolic disorders [15]. Bailing capsule (BL) (Cordyceps sinensis) has been approved by the State Food and Drug Administration of China (State Drug License Z10910036), and its chief ingredient is the fermentation product of *C. sinensis* (*Ophiocordyceps sinensis (Berk.)* G.H. Sungetal). BL contains various active ingredients, including cordycepin and its derivatives, alkaloids, sterols, trace elements, fatty acids, mannitol, and amino acids. These chemical components exhibit diversified physiological effects, such as antioxidant, antifibrotic, antitumor, antiviral, and anti-inflammatory effects [16, 17]. *C. sinensis* has been used widely as a herbal medicine and functional food for centuries. Several studies have established that *C. sinensis* upregulates steroidogenic enzymes and ovarian 17β-estradiol in human granulosa lutein cells and affects the quality of mature oocytes [18]. Clinical studies have shown that BL (*C. sinensis*) can reduce the abnormalities in lipid metabolism by regulating the PPARα pathway, enhancing lipolysis, and lowering the accumulation of renal triglycerides in diabetic rats [19]. Diane-35, when combined with BL, can improve glucose–lipid metabolism and IR in patients with PCOS and protect renal function without negative effects on hepatic function [20]. Moreover, this combination of metformin can improve endocrine dysregulation in patients with PCOS; regulate the levels of follicular fluids BMP-15, GDF-9, and IGF-1; promote the growth of oocytes and the formation of dominant follicles; and reduce the levels of serum sex hormones, such as luteinizing hormone and testosterone [21, 22]. In addition, it can reduce the levels of serum inflammatory factors, such as IL-6, TNF-α, APN, and LEP, thus effectively suppressing inflammation [23]. The combination of letrozole and BL can reduce ovarian volume, thicken the endometrium, and assuage the symptoms of hirsutism [24, 25]; however, the underlying mechanisms have not been adequately investigated.

Modern bioinformatics aids in exploring the mechanism of action of TCM. To identify the differential genes in clinical data samples, the GEO database was mined and analyzed using the R language. Subsequently, network pharmacology and molecular docking were used in a combined manner to link the active components of medications and target proteins and disease pathways to create a “drug–target–disease” relationship network. Sun et al. [26] investigated the mechanism of *Ocimum sanctum*–ginseng medication to prevent and treat diarrheal irritable bowel syndrome using gene expression integrated microarray data combined with network pharmacology. Wu et al. [27] employed the GEO database and TCGA differential analysis in conjunction with network pharmacology to predict the occurrence of gastric cancer. The completeness and systematic nature of this bioinformatics research strategy agree with the multicomponent, multimethod, and multitarget synergistic effects of Chinese medicine. Furthermore, this approach is in line with the holistic view theory of Chinese medicine and offers a novel way to investigate the mechanism of action of intricate Chinese medicine components.

In this study, PCOS mice models were constructed by feeding a high-fat and high-sugar diet combined with the subcutaneous injection of dehydrogenated epiandrosterone (DHEA) into the back of the neck [28]. The estrous cycle, ovarian pathological changes, and testosterone and estradiol sex hormone indexes of mice were observed to determine the efficacy of BL in improving PCOS. Next, to predict the pharmacological ingredients and multitarget mechanism of BL in treating PCOS, a combination of GEO chip analysis and network pharmacology was applied. Molecular docking and qPCR techniques were employed to validate the predicted results, and the system-level concept of multicomponent, multitarget, and multi-pathway actions of BL was elucidated. This research is expected to serve as a theoretical guide for further investigations into the mechanisms by which BL alleviates PCOS.

## Materials and methods

### Material and reagents

BL were purchased from Zhongmei Huadong Pharmaceutical Co., Ltd. (Hangzhou, Zhejiang, China); High-sugar and high-fat model feed (TP0800, 20% sucrose, 15% lard, 1.2% cholesterol, 0.2% sodium cholate, 1% bile salt, appropriate casein, calcium hydrogen phosphate, stone powder, etc.) was purchased from Nantong Trophy Feed Technology Co., Ltd. (Nanjing, Jiangsu, China); dehydrogenated epiandrosterone (DHEA) was purchased from Jiasheng Chemical Reagent Business Center (Shouguang, Shandong, China); Hematoxylin and Eosin (H&E) was purchased from Shanghai Yuanye Biotechnology Co., Ltd. (Shanghai, China), mouse serum testosterone T ELISA kit, and mouse serum estradiol E2 ELISA kit from Jianglai Biotechnology Co., Ltd. (Hangzhou, Zhejiang, China); SPARKeasy Improved Tissue/Cell RNA Kit, SPARKscript II RT plus Kit, and 2×SYBR Green qPCR Mix purchased from SparkJade (Shandong, China).

### Preparation and administration of PCOS model

**Fig. 1:**
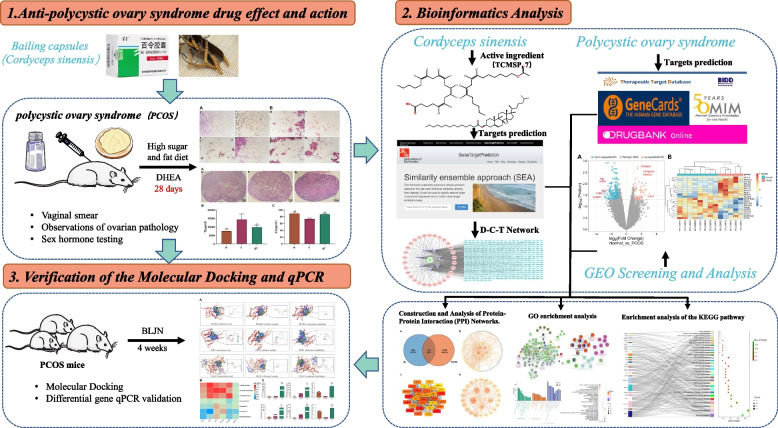
Experimental flowchart

Twelve SPF grade female ICR mice weighing 20 ± 5 g were obtained from Shanghai Slake Experimental Animal Co., Ltd. (Shanghai, China). The animals were maintained in controlled feeding conditions with a temperature range of 22–26 ℃, humidity between 45–50%, and a 12-h cycle of light and darkness. Water and feed were changed daily, and hygiene was maintained by regular cleaning. All animal procedures followed the “Regulations on the Management of Experimental Animals” and were approved by the Zhejiang University of Technology’s ethics committee. The experimental flow is shown in Fig. 1.

After 3 days of adapted feeding, ICR mice were divided into weight-based groups at random and given diets high in fat and sugar along with subcutaneous injections of exogenous androgens into the back of the neck for a total of 28 consecutive days, with the exception of the normal control group. Subsequently, vaginal smears were collected for four consecutive days, stained with H&E, and observed under the microscope. The mice in the model group showed obvious inhibition and disturbance of the motility cycle, suggesting that the modeling of PCOS mice was successful. At the end of modeling, the mice were divided into three groups according to their body weight: (1) normal group (NC); (2) model group (PCOS); (3) BL group. The dose was converted to the dose according to the body surface area coefficient and given by gavage. Normal and model control mice were given purified water by gavage at a dosing volume of 1 mL/100 g. BL was configured to a concentration of 240 mg/mL and administered by gavage once daily for 4 weeks. At the end of the experiment, mice were fasted overnight and euthanized by anesthesia with 0.3% pentobarbital sodium solution (0.2 ml/10 g, IP). Serum was obtained via orbital blood collection, ovarian tissue was collected, washed with cold saline, and stored at -80 ℃.

### Estrous cycle monitoring

Vaginal smears were obtained from mice beginning on day 28 of modeling and stained with H&E for 4 days in a row. The morphology and coloration of cells were observed under a biological microscope using the following criteria: Proestrus (predominantly oval nucleated epithelial cells with a few keratinized epithelial cells), Estrus (a large number of keratinized epithelial cells in the form of deciduous stacks), Metestrus (similar numbers of keratinized epithelial cells, leukocytes, and nucleated epithelial cells), and Diestrus (predominantly leukocytes with a few epithelial cells and mucus). The presence of an inhibited or disturbed estrous cycle indicates successful modeling. After 4 weeks of administration, vaginal smears were observed for 8 consecutive days to analyze changes in the mice’s estrous cycle.

### Observation of hematoxylin and eosin (H&E) staining in ovarian tissues

The ovaries were embedded in paraffin after being treated in 4% formalin. All specimens were then cut into sections with a 4μm thickness and stained with hematoxylin and eosin (H&E). Finally, tissue images were captured using a biomicroscope.

### Serum sex hormone analysis

An orbital blood sampling technique was used to gather blood samples. The samples were then incubated for 30 min at 37 °C, followed by 10 min of centrifugation at 3000 rpm at 4 °C. After removing the supernatant, the procedure above was carried out one more. For upcoming investigations, the serum was collected in 1.5 mL Eppendorf tubes and kept at -80 °C.

### Screening of the active ingredients and target of BL

The Traditional Chinese Medicine Systems Pharmacology Database and Analysis Platform (TCMSP) was utilized to collect the chemical constituents of BL. This was supplemented by the results of CNKI and Pubmed literature searches. TCMSP is a systematic pharmacology platform for TCM that provides information on TCM components, compounds, and pharmacokinetic effects of natural compounds [29]. It includes drug similarity, oral bioavailability, intestinal epithelial permeability, water solubility, and blood-brain barrier permeability. The screening criteria for bioactive compounds were as follows: oral bioavailability (OB) ≥ 30% and drug-likeness (DL) ≥ 0.18 [30]. OB refers to the ability of an orally administered drug to be delivered to the body circulation [31]. DL is based on similarity to the functional groups and physical properties of known drugs [32]. Further analysis was carried out on compounds that met the OB and DL thresholds as active compounds [33]. The TCMSP database’s active components were then searched with a Probability > 0.1 in Swiss Target Prediction [34] and the SEA database [35]. The probable targets of BL were determined by combining the targets from the three databases, eliminating duplicate values, and standardizing the species to “Human” utilizing the UniProt database [36].

### Construction of PCOS-related target database

The keyword “polycystic ovary syndrome” was used to search for samples in NCBI’s GEO database. Studies with a sample size greater than 10 were then screened, and four datasets (GSE1615, GSE5090, GSE5850, and GSE48301) containing disease samples and normal samples were selected as the study population. Subsequently, the samples were subjected to principal component analysis and clustering hierarchical analysis to evaluate the acceptable quality of each set of samples. Systematic analysis was performed by R 4.2.3, with joint analysis and batch correction using the SVA and Limma packages, with a threshold of |logFC|**≧**1 and an adjusted *P*-value < 0.05 for differential gene identification [37]. Volcano and heat maps were visualized using the ggplot2 and pheatmap packages in R 4.2.3 [38, 39]. The keyword “polycystic ovary syndrome” was used to search from GeneCards database [40], DrugBank database [41], OMIM database [42] and TTD database [43]. Finally, the genes obtained above were combined and duplicates were deleted to establish the disease target database.

### Construction of “drug–component–target” modulation network

The objectives and active components gathered in the preceding phase were adjusted. The software Cytoscape 3.8.2 was used to map the drug–component–target modulation network after the network files and annotation files had been loaded.

### Building and examining PPI networks

Targets connected to PCOS were used to map the prospective targets of BL. Venn diagrams were generated using TBtool software to identify cross-targets as regulatory targets of BL against PCOS [44]. The STRING PPI database then imported these targets with default values for each option [45]. Isolated targets were hidden to obtain protein interaction networks, which were exported to CSV format. Using the CytoHubba plugin of Cytoscape 3.8.2 software, hub genes were identified. The MCC algorithm is considered the most effective method for finding the key nodes in the co-expression network [46–48]. The MCC algorithm sets the parameter by selecting the top 30 ranked targets and filtering them based on the degree value > 30. The resulting target is then designated as the core gene.

### Analysis of KEGG pathways and GO enrichment

The core genes were analyzed for Gene Ontology (GO) enrichment using the Metascape database with the species set as H. sapiens, applying FDR < 0.05 and *P* < 0.05 as the critical values for GO Molecular Functions, Biological Processes, and Cellular Components [49]. The Kyoto Encyclopedia of Genes and Genomes (KEGG) enrichment analysis [50–52] was performed using the Visualization and Integrated Discovery (DAVID) database, with species selected as Homo sapiens [53]. The critical values for the analysis were set at FDR < 0.05 and *P* < 0.01, while the default values for other parameters were used. The visualization was plotted by https://www.bioinformatics.com.cn (last accessed on 10 Nov 2023), an online platform for data analysis and visualization [54].

### Composition and molecular docking of the target

Based on the results of network pharmacology and literature research, potential pathways of action were identified. Molecular docking was then performed for validation. The 2D structure of the matching active component was retrieved from the NCBI database, and the 3D structure of the protein target was obtained from the PDB database. SYBYL-X software was used for molecular docking with the Surflex-Dock Geom docking mode. The default number of dockings was set to 20. The total score was shown on a heat map, and the docking score was used to assess the binding capacity and activity of the target and active substance. The optimal result for ligand-receptor binding was determined by the binding mode with a Total score > 7 [55].

### Detection of PCOS gene expression by real-time fluorescence quantitative PCR

Total RNA from mouse ovary tissues was extracted with SPARKeasy Improved Tissue/Cell RNA Kit (SparkJade, Shandong, China) and reverse-transcribed using 2 × SPARKscript II RT Plus Master Mix (SparkJade, Shandong, China) according to the manufacturer’s instructions. The primers used were synthesized by SparkJade (Shandong, China) (Table 1). And real-time quantitative (qRT) PCR was done using 2×SYBR Green (SparkJade) in the ABI 7500 system (ABI, USA). The PCR cycling profile is one cycle at 94 °C for 3 min, 40 cycles at 94 °C for 10 s, and 60 °C for 34 s.

**Table 1:** Primer sequence

**Genes**	**Sequences**	**Gene ID**
JAK2	FORWARD: CGAAGCAGCAAGCATGATGAGTC	NM_001048177.3
	REVERSE: GTTCTCCTCTCCACAGACACAGAC	
PPARG	FORWARD: TGTTCGCCAAGGTGCTCCAG	NM_001127330.3
	REVERSE: TGAAGGCTCATGTCTGTCTCTGTC	
PI3K	FORWARD: GGGAGCAGCAACCGAAACAAAG	NM_001024955.2
	REVERSE: CCACTACGGAGCAGGCATAGC	
AKT1	FORWARD: AAGCGGACGCTTCACGAATTTG	NM_001165894.2
	REVERSE: ATCCAGTGCAGGGTCCGAGG	
IRS1	FORWARD: CAGTGGATGGCAGTCCTGTGAG	NM_010570.4
	REVERSE: CAGTGGATGGCAGTCCTGTGAG	
ESR1	FORWARD: GCCAAGGAGACTCGCTACTGTG	NM_001302531.1
	REVERSE: CAGCCTTCGCAGGACCAGAC	
β-actin	FORWARD: GATGGTGGGAATGGGTCAGAAGG	NM_007393.5
	REVERSE: TTGTAGAAGGTGTGGTGCCAGATC	

### Statistical analysis

The bar charts in the study were produced using GraphPad Prism 6.0 and analyzed using SPSS 20.0 for one-way ANOVA. Results were expressed as mean ± standard deviation ($\overline{\mathrm{x}}\pm \mathrm{SD }$). The t-test was used to compare the mean of two samples for intergroup differences, with statistical significance set at *p* < 0.05.

## Results

### BL improve estrous cycle and ovarian dysfunction in PCOS mice

**Fig. 2:**
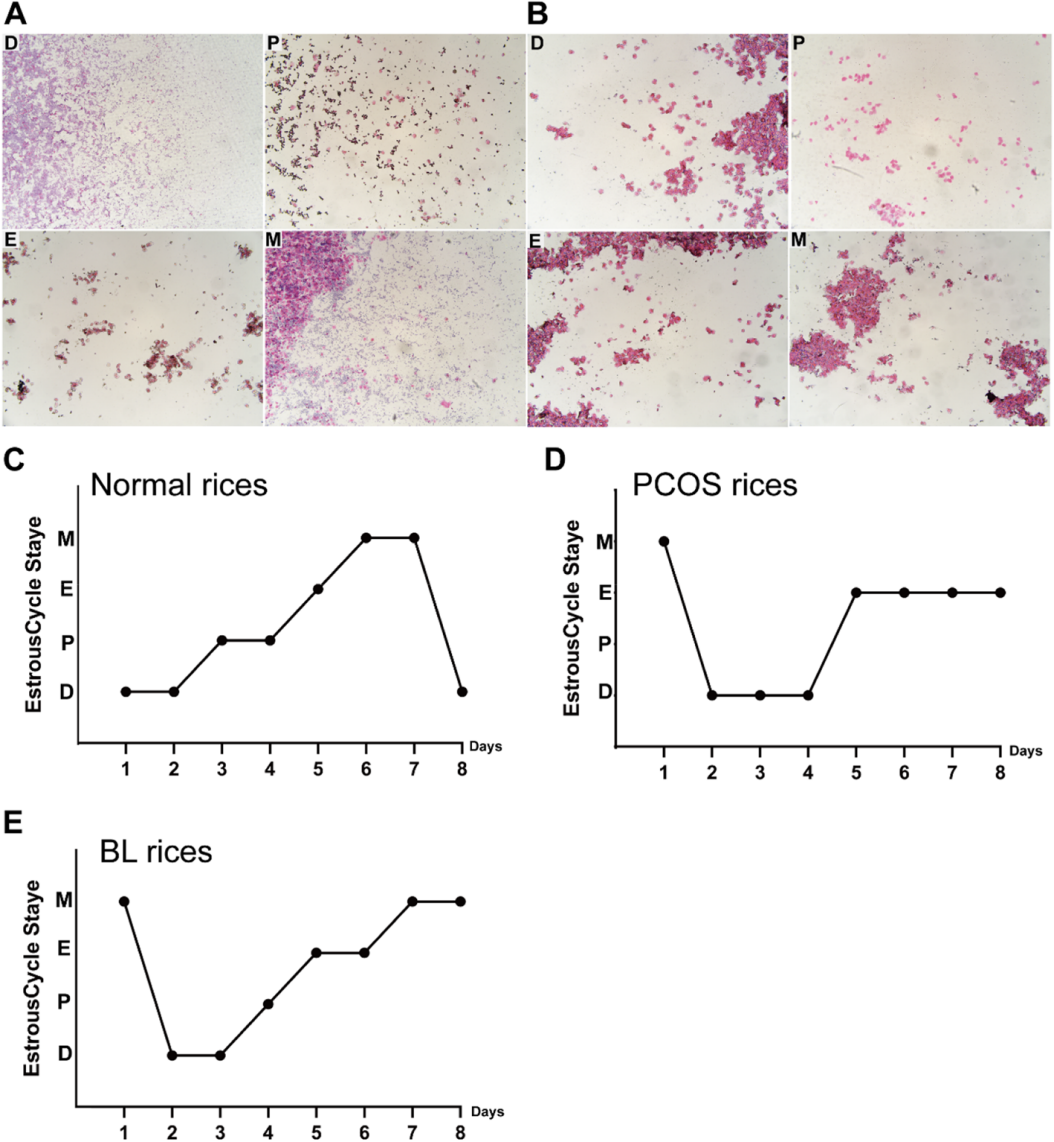
Changes in the estrous cycle of mice. **A** The four stages of the estrous cycle in normal mice; **B** The four stages of the estrous cycle in PCOS mice; **C**-**E** Changes in the estrous cycle of mice in the normal group, model group, Bailing capsule group. P: Proestrus, E: Estrus, M: Metestrus, D: Diestrus

Estrous cycle monitoring for 4 days after modeling to confirm the presence of typical PCOS-like alterations in the modeled mice. Cytological analysis of vaginal smears indicated a disturbed estrous cycle in DHEA-induced mice, as shown in Fig. 2A and B, where the four phases of the estrous cycle were depicted for normal and PCOS mice. The estrous cycle assessment demonstrated significant changes in the PCOS group compared to the normal mice, with most alterations occurring during the estrous phase (Fig. 2C and D). Estrous cycle disorder was improved after treatment with BL (Fig. 2E).

**Fig. 3:**
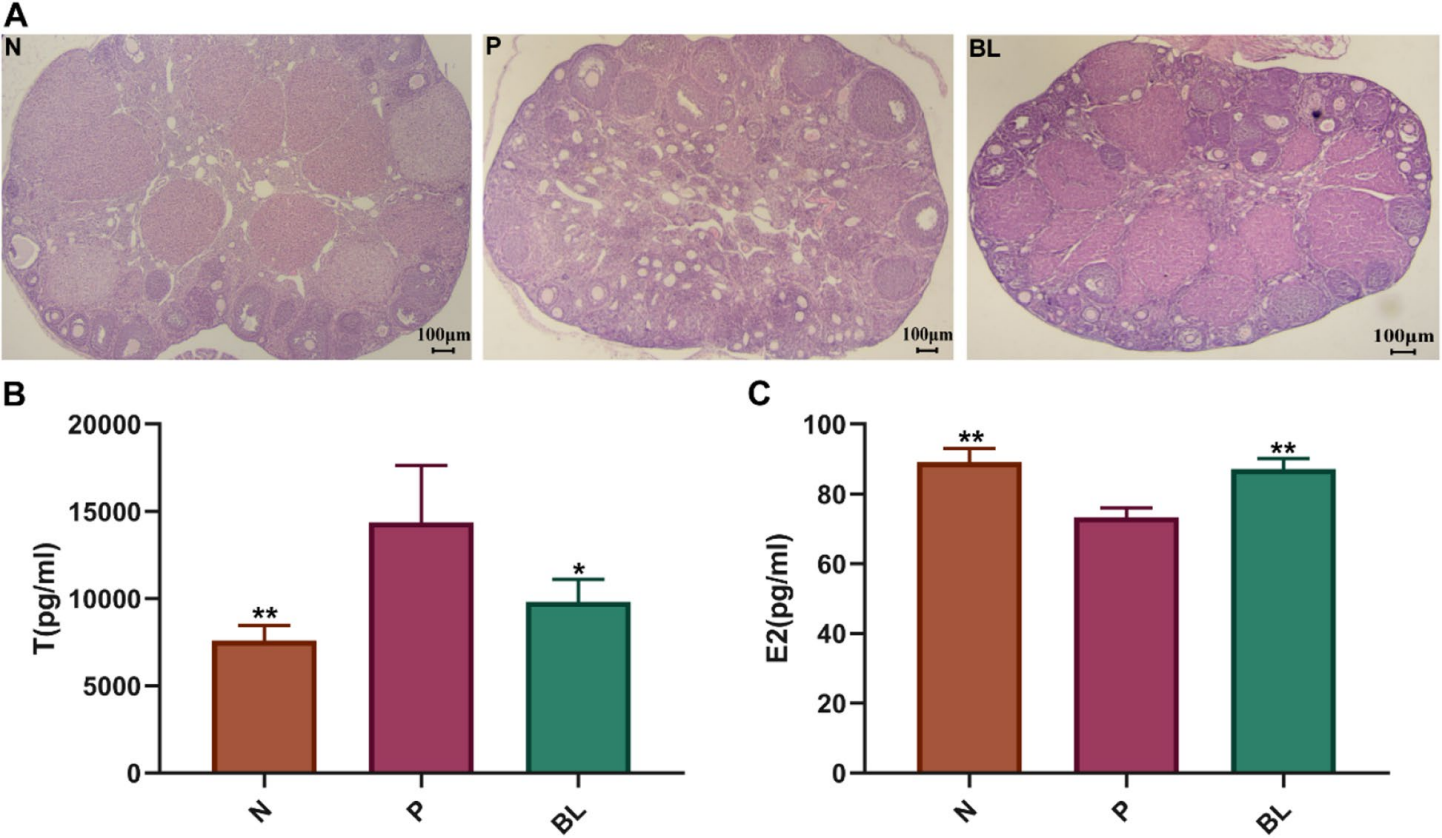
Effect of supplementation with Bailing capsule on ovarian dysfunction and serum sex hormone levels in DHEA-induced PCOS-like rats. Serum hormonal levels were using enzyme linked immunosorbent assay (ELISA). **A** Section of the ovary from each experimental group (H&E, scale bar = 100 µm). **B** Testosterone(T), **C** Estradiol(E2). All values represent means ± SD, *n* = 4 per group. *p < 0.05, **p < 0.01 vs PCOS. N: normal group N: normal group; P: PCOS group; BL: Bailing capsule group

We further utilized H&E staining on ovarian tissues to validate these findings. Compared with the normal group, the ovarian morphology of PCOS mice was altered, exhibiting thickened ovarian cortex, a heterogeneous structure, few or no corpus luteum, cystic follicles covered by several layers of granulosa cells or follicular membrane cells, many enlarged cystic oocysts, and ovaries showing typical polycystic changes with a marked reduction in the number of primordial and major follicles (Fig. 3A). There was a significant improvement in ovarian symptoms in the BL group (Fig. 3A). Our findings suggest that BL improved the pathological damage to ovarian tissue in PCOS-like mice.

### BL regulate serum sex hormone levels in DHEA-induced PCOS-like mice

Apart from the observed alterations in ovarian tissue, treatment with BL also led to significant changes in serum T and E2 levels in PCOS-like mice. As illustrated in Fig. 3B and C, DHEA-induced PCOS-like mice exhibited significantly elevated serum T (*p* < 0.01) and decreased E2 levels (*p* < 0.01) compared with normal mice. However, administration of BL resulted in significantly lower serum T levels and higher E2 concentrations relative to the PCOS group (*p* < 0.05).

### Active ingredients and targets of BL

By applying the TCMSP database and literature search, a total of 38 chemical components of BL were found. These 38 chemical components were then further screened under the circumstances of OB ≥ 30% and DL ≥ 0.18 to find a total of 7 active components of BL (Table 2). The aforementioned active ingredients were looked up in the Swiss Target Prediction and SEA databases. The targets with Probability > 0.1 were screened, and duplicate values were eliminated. The protein targets were then normalized to gene targets by the Uniprot database to obtain 437 corresponding targets.

**Table 2:** Information on the active ingredients of Bailing capsules

**Mol ID**	**Molecule Name**	**Structure**	**MW**	**OB (%)**	**DL**
MOL001439	arachidonic acid		304.52	45.57	0.20
MOL000358	beta-sitosterol		414.79	36.91	0.75
MOL008998	cerevisterol		432.76	39.52	0.77
MOL000953	cholesterol		386.73	37.87	0.68
MOL008999	cholesteryl palmitate		625.19	31.05	0.45
MOL001645	linoleyl acetate		308.56	42.10	0.20
MOL011169	peroxyergosterol		428.72	44.39	0.82

### Active drug-ingredient-target network construction of BL

**Fig. 4:**
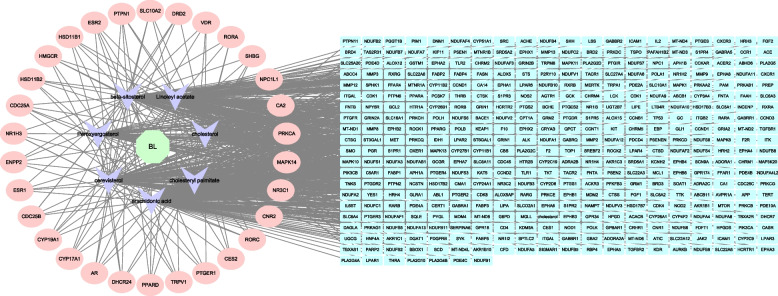
D-C-T network. The green hexagon is the drug; the purple V-shape is the active ingredient; the circle is the target with degree > 2, indicating more than two active ingredients acting on this target; the rectangle is the target with degree = 1

We imported data on the 7 active ingredients and their targets for BL into CytoScape 3.8.2 software to develop a visualization diagram of the drug targets of these active ingredients, as shown in Fig. 4. The primary active ingredients include Linoleyl acetate, cholesteryl palmitate, arachidonic acid, cerevisterol, and beta-sitosterol. These compounds exhibit numerous corresponding targets, including AR, PPARD, PTPN1, ESR1, ESR2, HSD11B1, HMGCR, MAPK14, CDC52A, NR1H3, and ENPP2.

### Construction of targets for interaction between the active ingredients of BL and PCOS disease

**Fig. 5:**
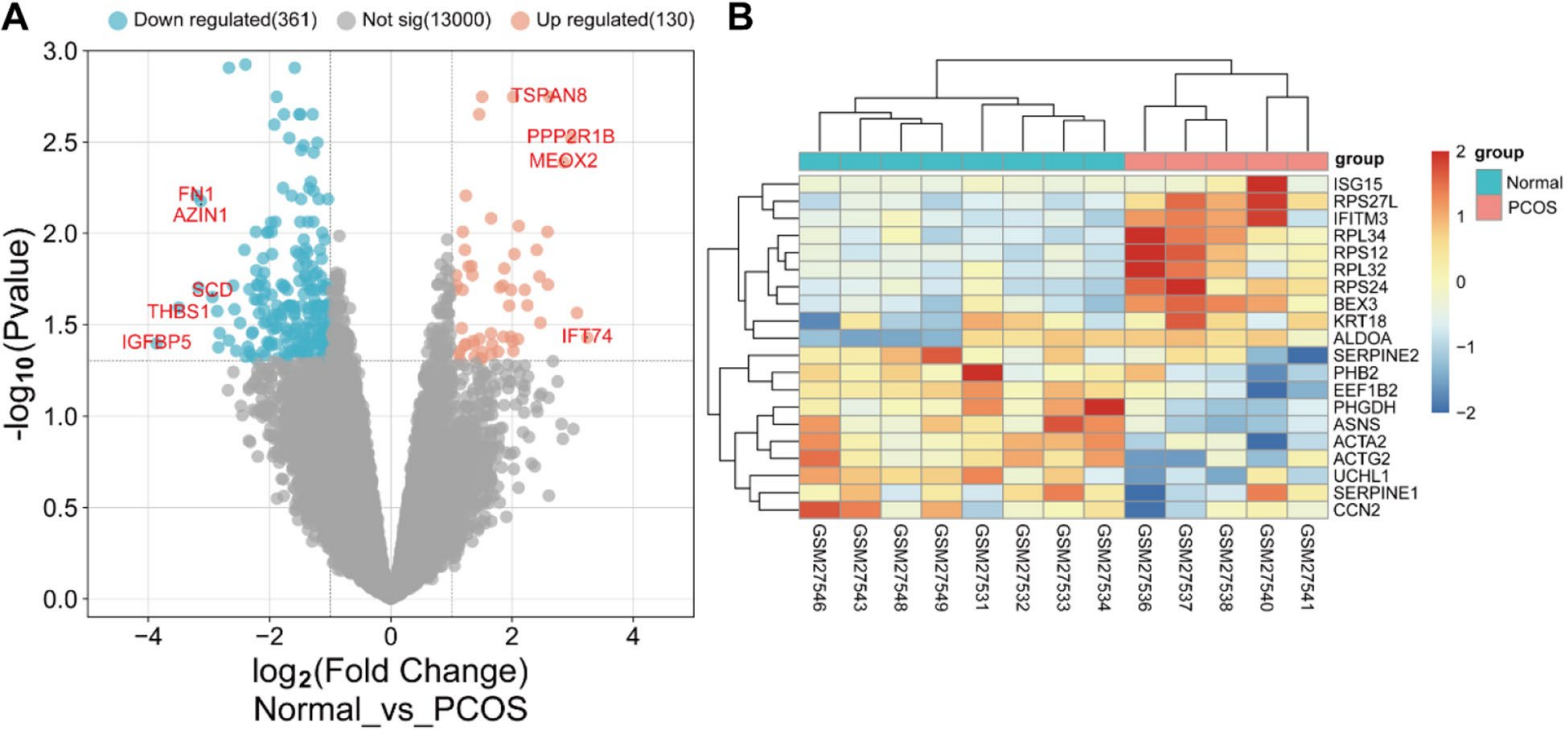
Heat map and volcano map of differential genes of Normal and PCOS patients with GSE1615 chip. **A** Volcano map. The X-axis was log2 (fold change). The Y-axis was -log10 (Pvalue). **B** We selected the top 20 differentially expressed genes for the heat map (blue is a low expression, yellow is a medium expression, and red is a high expression). All genes were first set as undifferentiated genes (denoted in Grey) and screened according to the logFC and adjusted *p*-value. When the adjusted *p*-value was < 0.05 and the logFC ≥ 1, it was noted as an upregulated gene (shown in red); when the adjusted *p*-value was < 0.05 and the logFC ≤ -1, it was noted as a downregulated gene (shown in blue)

**Fig. 6:**
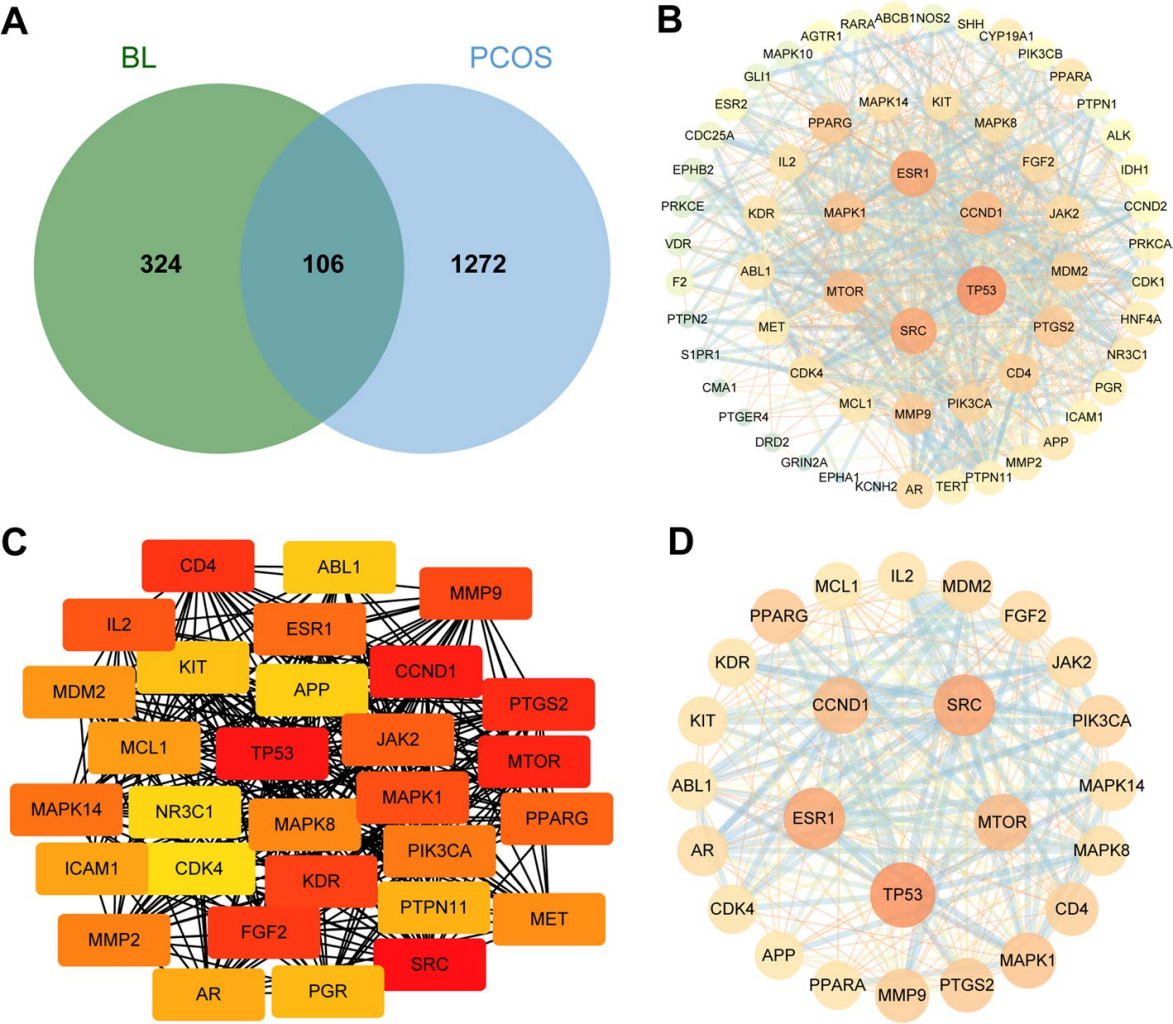
Construction and analysis of protein-protein interaction (PPI) networks. **A** Venn diagram of shared targets of BL and PCOS. Blue circles represent BL targets, orange circles represent disease targets. **B** PPI network from database. The circle represents the target point, the larger the radius of the circle, the darker the fill color means the larger the target point degree, the darker and thicker the line between the target points means the larger the combine score. **C** MCC algorithm. The darker the color, the higher the value of the target. **D** Degree algorithm. The darker the color, the higher the value of the target

Cluster analysis and PCA analysis of the four sample sets showed that the quality of the GSE1615 microarray samples was suitable for subsequent analysis. 491 DEGs were identified, comprising 130 up-regulated genes and 361 down-regulated genes. As shown in Fig. 5, the volcano and heat maps of these 491 DEGs. The total number of targets obtained from various databases was 1096, and after eliminating duplicates and integrating disease targets from GeneCards, DrugBank, OMIM, and TTD databases, a total of 1378 PCOS disease targets were identified. The intersection of BL active compound targets with PCOS disease targets revealed 106 common targets, which are crucial for the anti-PCOS activity of BL compounds (Fig. 6A).

### PPI network construction

To investigate the mechanism of therapeutic action of BL on PCOS, we imported 106 shared targets into the STRING database to construct a PPI network. The number of edges of this network was 1265, and the average degree value of targets was 17.6 with PPI enrichment *p*-value < 1.0e-16 (number of edges: 1265, average node degree: 17.6, PPI enrichment *p*-value: < 1.0e-16). We identified core PPI networks based on topological analysis using median ≥ 2× as a screening criterion. As shown in Fig. 6B, the node size in the network is proportional to the degree of targeting. The MCC of each node was calculated by the plug-in cytohubba in Cytoscape, and the top 30 were selected as the key genes (Fig. 6C), followed by network analysis using the network analysis tool. In the network, the targets with degree value ≥ 30 in the top30 genes were further screened, and a total of 27 targets were obtained and used as the key targets (Fig. 6D), namely TP53, SRC, ESR1, MTOR, CCND1, MAPK1, PPARG, PTGS2, MMP9, CD4, PIK3CA, MDM2, FGF2, JAK2, MAPK8, AR, MAPK14, KIT, CDK4, IL2, PPARA, KDR, ABL1, APP, TLR4, NR3C1, MCL1. These targets may represent the core targets for the action of BL.

Several predicted targets identified in this study are related to various biological processes, including glycolipid metabolism (PPARG, PIK3CA, PPARA), inflammatory response (SRC, ESR1, MAPK1, PTGS2, MAPK8, MAPK14), immunomodulation (CD4, IL2), and hormone regulation (AR, JAK2). These findings suggest that the therapeutic effects of BL may involve multiple mechanisms, including modulation of inflammatory responses, regulation of glycolipid metabolism, immunomodulation, and hormone regulation.

### Analysis of GO functional enrichment

**Fig. 7:**
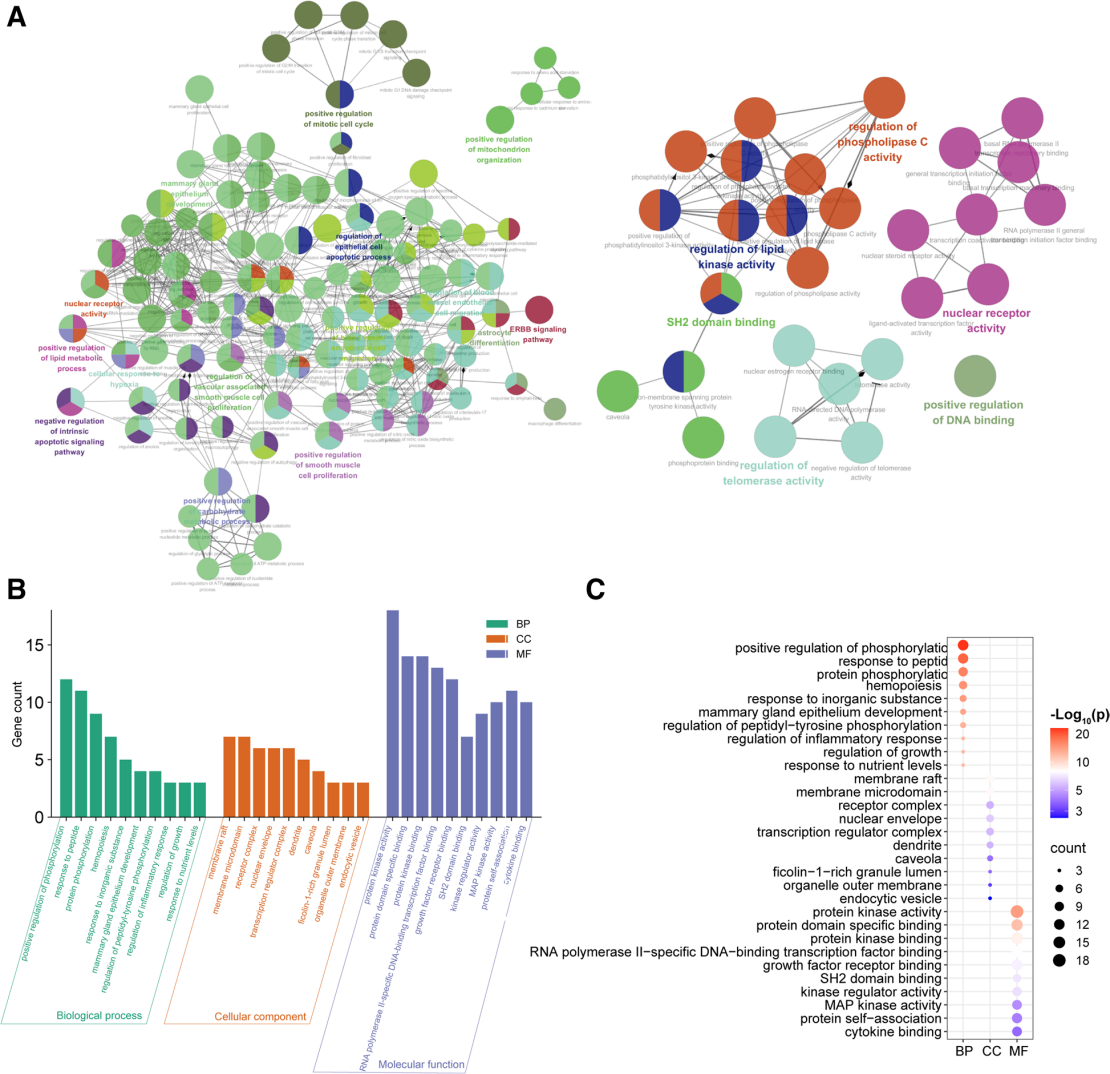
GO enrichment analysis. **A** GO enrichment networks. **B** Go enrichment bar plot. Different colors indicate different types of enrichment, and the higher the bar indicates the higher the number of genes in that enrichment term. **C** Go enrichment dotplot. The size of the bubbles indicates the number of gene enrichment, and the darker the red color indicates the larger the *p*-value

To explore the various mechanisms of BL involvement in the treatment of PCOS, we obtained 642 BF entries, 37 CC entries, and 46 MF entries through the Metascape database. The results revealed that BP was mainly involved in positive regulation of phosphorylation, protein phosphorylation, and response to peptide. MF was mainly involved in protein kinase activity, protein kinase binding, and protein domain-specific binding. Additionally, CC was mainly associated with caveola, nuclear envelope, and transcription regulator complex. To visualize the GO enrichment network, ClueGo was utilized (Fig. 7A), followed by enrichment analysis bar charts (Fig. 7B) and dotplot (Fig. 7C) for the top 10 ranked data based on the composite scores.

### Analysis of the KEGG pathway’s enrichment

**Fig. 8:**
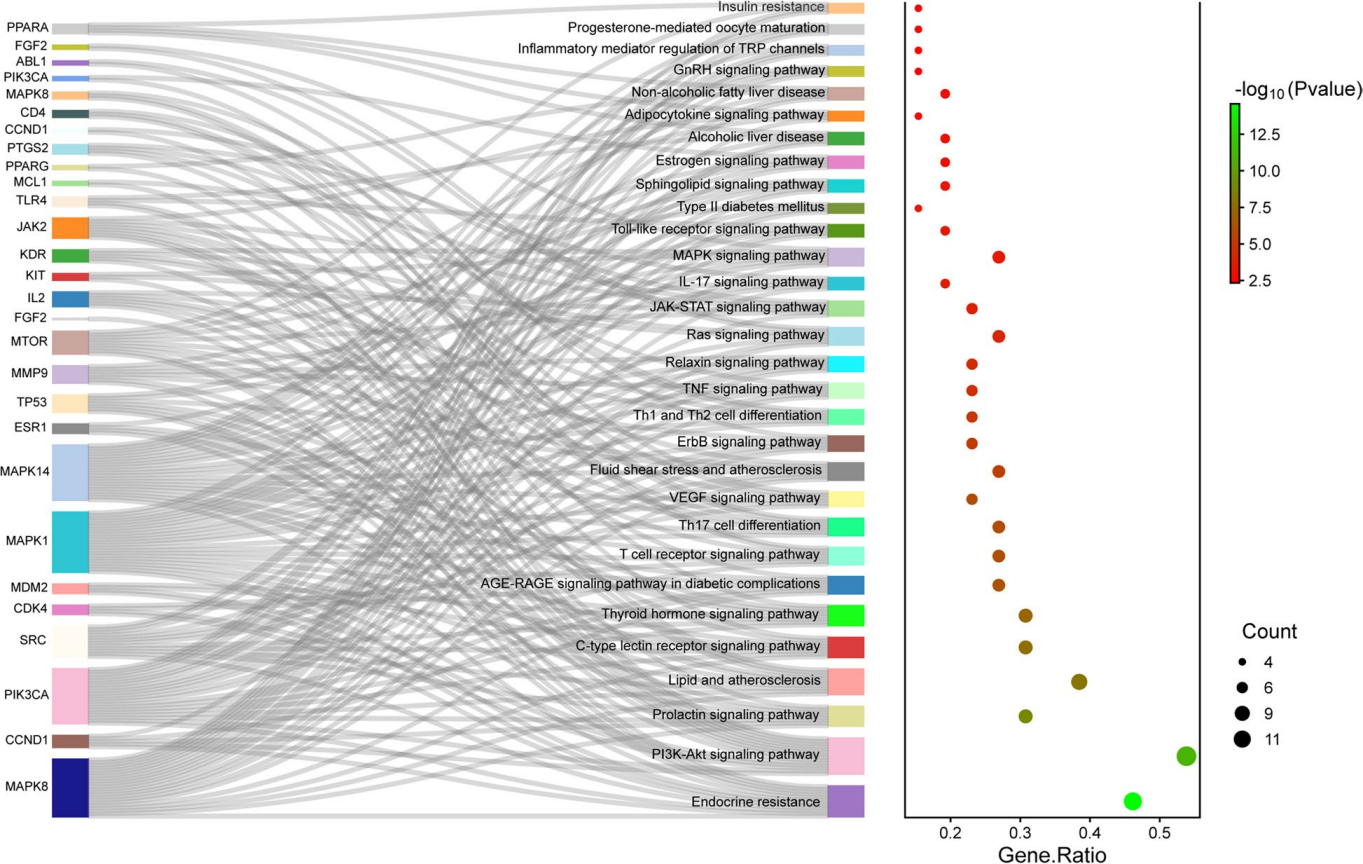
Enrichment analysis of the KEGG pathway. On the left is a Sankey plot, reflecting the enrichment of genes with respect to the pathway. On the right is the bubble plot, the size of the dots indicates the number of genes in the pathway, the X coordinate indicates the Gene Ratio, and the color represents the pathway enrichment-log10 (Pvalue), with red indicating a larger value and green indicating a smaller value

The KEGG enrichment analysis of the intersecting targets in the PPI network of BL and PCOS was performed using the DAVID database. A total of 112 pathways with *p*-value < 0.01 were obtained, and after deleting cancer, viral infection, and other pathways unrelated to PCOS, pathways with the number of genes numbered for enrichment of the number of pathways ≥ 4 were selected. Sankey plot and bubble plots were created using R 4.2.3 (Fig. 8). According to our research, BL may be useful in treating endocrine resistance, PI3K-Akt signaling pathway, prolactin signaling pathway, thyroid hormone signaling pathway, TNF signaling pathway, estrogen signaling pathway, Toll-like receptor signaling pathway, insulin resistance, and other pathways that are involved in the regulation of PCOS.

### Verification of the molecular docking

**Fig. 9:**
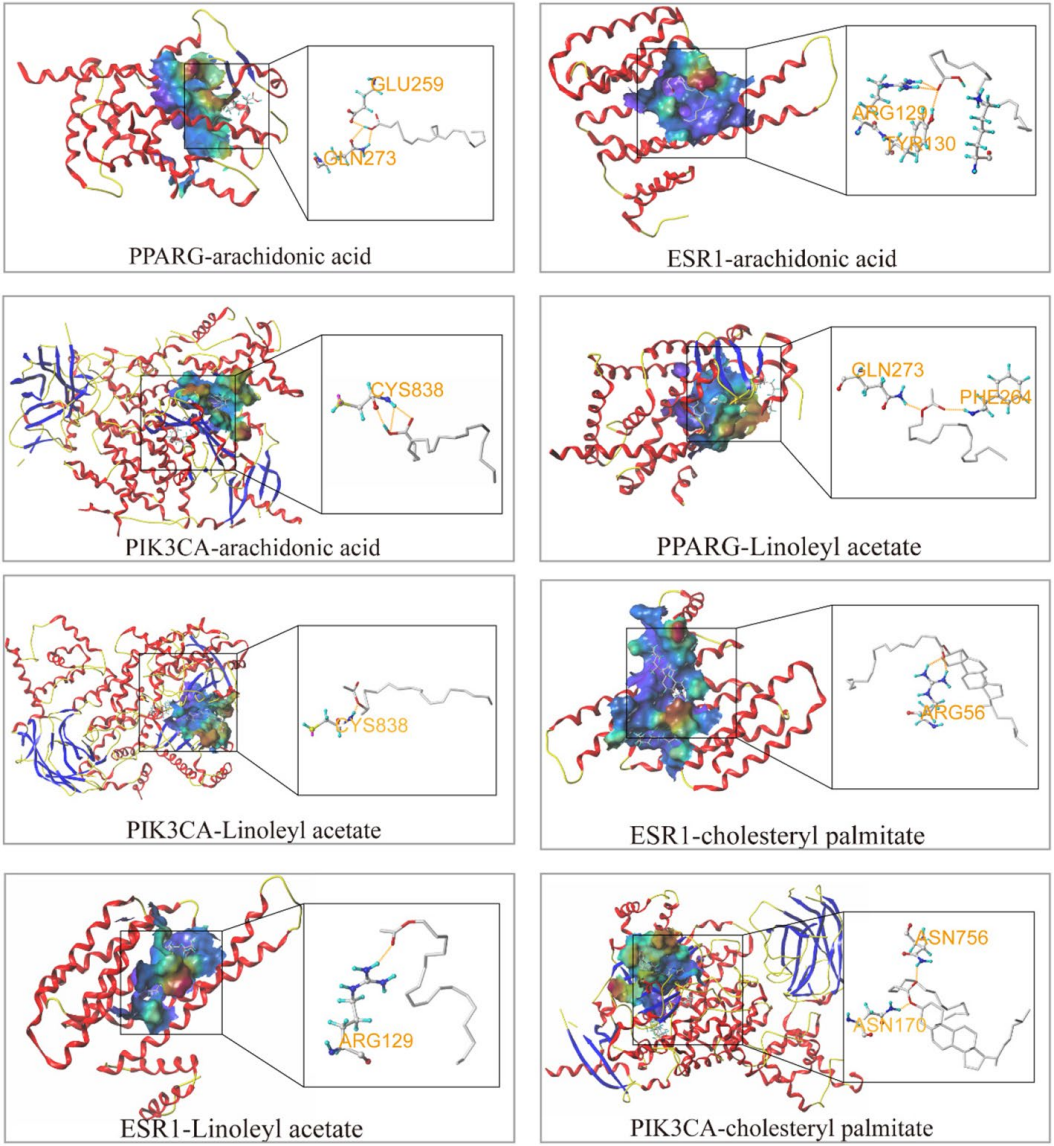
Molecular docking. Visualization of partial molecular docking results. The yellow dashed line represents the polar connection of the active ingredient to the target, the small molecule in gray is the active ingredient, and the amino acid residues connected to the small molecule are represented by the rod model

**Fig. 10:**
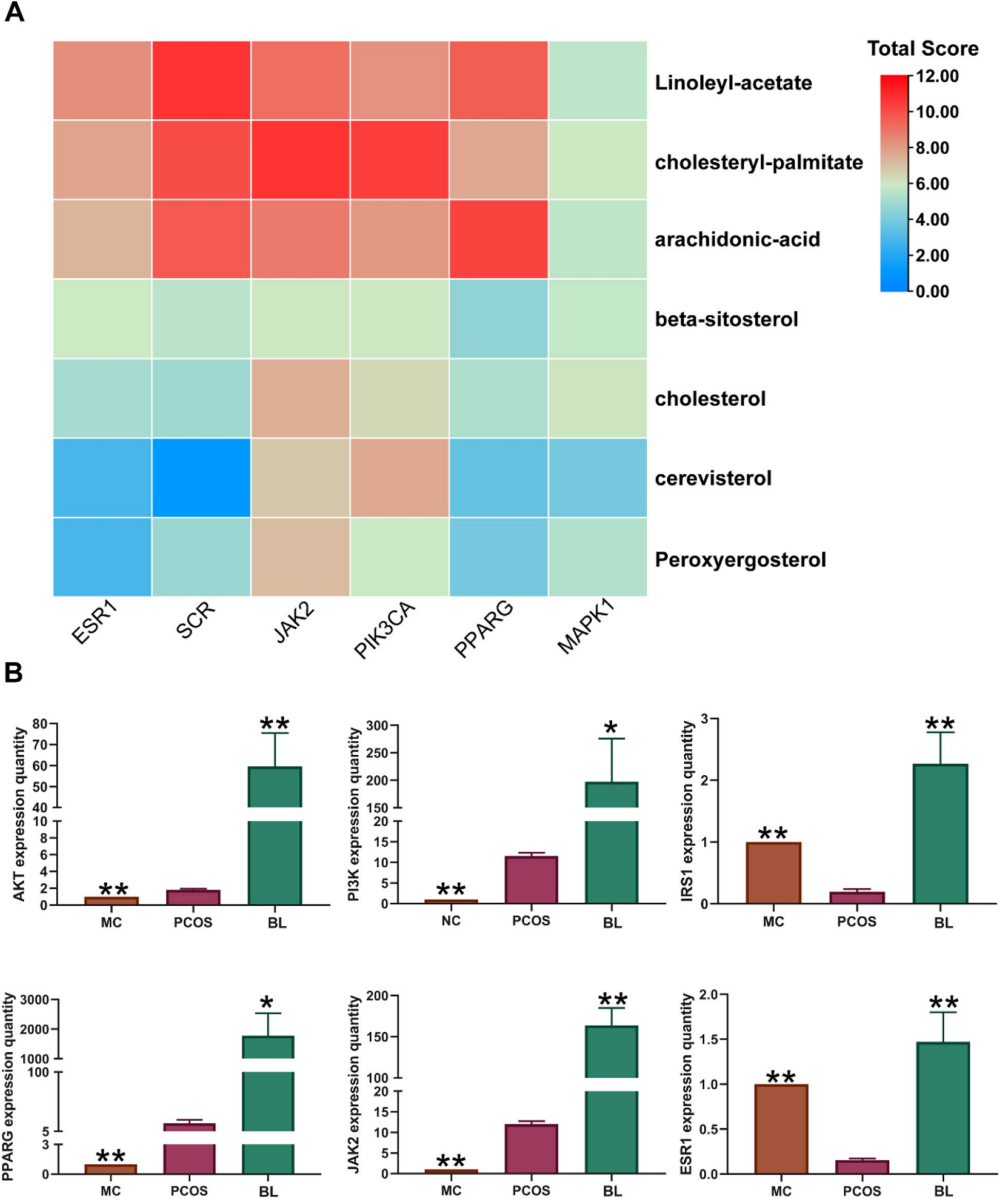
qPCR validation. **A** Total score heat map. The darker the red color, the higher the docking score of the component and the target. **B** Real time fluorescence quantitative PCR detection of the relative expression level of PCOS gene mRNA. All values represent means ± SD, *n* = 4 per group. *p < 0.05, **p < 0.01 vs PCOS

In this study, we used SYBYL-X software to rank the top 7 compounds (linoleyl-acetate, cholesteryl palmitate, arachidonic acid, beta-sitosterol, cholesterol, cerevisterol, and peroxyergosterol). We interlinked the compounds with the 6 core targets (SRC, ESR1, MAPK1, PPARG, PIK3CA, and JAK2) one by one and determined their matching degree based on Total Score, Crash, and Polar (Table 3). A heat map was generated based on the Total Score (Fig. 10A). Typically, a Total Score ≥ 7 often denotes great docking, whereas a score ≥ 3 denotes fair docking. We selected targets related to the Toll-like receptor signaling pathway and insulin resistance with the highest Total Score for the presentation of representative maps of important target-active component docking patterns (Fig. 9). The results showed that arachidonic acid, linalool acetate, and cholesteryl palmitate had good binding with the predicted targets.

**Table 3:** Molecular docking results

Target	Target (PDB ID)	Target structure	Compound	TotalScore	Crash	Polar
ESR1	7BAA		Linoleyl acetate	8.3251	-0.9671	1.8737
			Cholesteryl palmitate	7.7857	-1.4484	1.5698
			Arachidonic acid	7.3428	-1.5082	2.3671
			Beta-sitosterol	5.9472	-0.4759	1.1488
			Cholesterol	5.0026	-1.3359	1.3577
			Cerevisterol	2.8532	-2.9468	2.7649
			Peroxyergosterol	2.8468	-3.9172	1.1674
SCR	7NG7		Linoleyl acetate	10.7475	-1.4934	1.1177
			Cholesteryl palmitate	10.0593	-2.3824	1.0774
			Arachidonic acid	9.7387	-1.2446	2.2609
			Beta-sitosterol	5.534	-1.7157	1.4174
			Cholesterol	4.8613	-2.5563	1.1546
			Cerevisterol	1.1292	-10.2949	2.094
			Peroxyergosterol	4.7896	-1.4452	0.0031
JAK2	3UGC		Linoleyl acetate	9.1788	-0.6585	1.0985
			Cholesteryl palmitate	10.7189	-1.9226	1.1604
			Arachidonic acid	8.88	-1.5142	4.202
			Beta-sitosterol	6.0178	-1.5458	0.9794
			Cholesterol	7.487	-1.7915	1.3683
			Cerevisterol	6.8316	-1.4211	1.3597
			Peroxyergosterol	7.1444	-0.7567	1.3823
PIK3CA	7JIU		Linoleyl acetate	8.2547	-0.8585	1.1105
			Cholesteryl palmitate	10.453	-1.8973	1.1969
			Arachidonic acid	8.133	-0.5848	2.1951
			Beta-sitosterol	5.9831	-1.5696	0.9443
			Cholesterol	6.4201	-1.1136	0.0312
			Cerevisterol	7.6627	-1.7372	2.8771
			Peroxyergosterol	5.8684	-1.1701	0.62
PPARG	6MS7		Linoleyl acetate	9.6126	-2.6586	1.7291
			Cholesteryl palmitate	7.714	-2.5063	1.1852
			Arachidonic acid	10.321	-2.9074	1.933
			Beta-sitosterol	4.5381	-5.0033	0.8153
			Cholesterol	5.2478	-1.4658	0.004
			Cerevisterol	3.5104	-4.1487	0.0116
			Peroxyergosterol	3.9174	-2.8043	1.1204
MAPK1	8AOJ		Linoleyl acetate	5.5979	-0.8926	1.6518
			Cholesteryl palmitate	6.0008	-1.342	0
			Arachidonic acid	5.6337	-1.7517	1.1426
			Beta-sitosterol	5.709	-1.7267	1.2469
			Cholesterol	6.1031	-1.2571	1.0163
			Cerevisterol	3.9207	-1.32	2.8704
			Peroxyergosterol	5.3368	-1.1662	1.6262

### Detection of mRNA expression levels of differential genes in PCOS

The mRNA expression levels of PCOS-related genes were determined using real-time fluorescence quantitative PCR assay. The results showed that *JAK2, PPARG, PI3K*, and *AKT1* gene expressions were significantly upregulated (*p* < 0.01), while *ESR1* and *IRS1* gene expressions were significantly downregulated (*p* < 0.01) in the PCOS model group compared with the normal group. These findings were consistent with the differential gene changes observed in microarray GSE1615 dataset. Additionally, compared to the model group, the BL group exhibited significant changes in gene expression, which were statistically significant (*p* < 0.05, *p* < 0.01) (Fig. 10B).

## Discussion

PCOS is characterized by androgen overproduction and ovarian dysfunction. This condition is categorized into PCOS in adolescence and PCOS in reproductive age, with the latter being more complex, more heterogeneous, and more prevalent among younger women. PCOS is the most common cause of infertility in women of reproductive age and accounts for 30–60% of all patients with infertility who exhibit ovulatory disorders [56]. Moreover, PCOS tends to co-occur with metabolic syndrome. The incidence of PCOS in women is estimated to be approximately 5%–15%; 70% of the patients with PCOS have abnormal lipid metabolism, of which approximately 41% develop nonalcoholic fatty liver disease [57, 58].

The pathogenesis and etiology of PCOS are yet to be elucidated, but emerging evidence hints that it may be a complex polygenic disease influenced by epigenetic and environmental factors, such as diet and lifestyle. However, IR has been reported to be a significant pathological basis for the development of PCOS [5]. A study has demonstrated that up to 44%–85% of patients with PCOS, particularly those who are obese, have IR [59]. When IR occurs, the body compensates by secreting excessive insulin, which leads to endocrine and metabolic disorders and exacerbates disease progression. Insulin sensitizers, such as metformin and pioglitazone, and other hypoglycemic drugs can improve IR. The clinical use of these drugs can significantly improve menstrual cycle regularity, reduce androgen levels, restore ovulation, enhance pregnancy rates and outcomes, and augment the treatment efficacy [60, 61].

Hyperandrogenemia is the central factor in treating PCOS as it can lead to ovarian stromal hyperplasia, thickening of the envelope, acceleration of follicular atresia, and ultimately, female ovulatory infertility. Excessive insulin secretion triggered by IR can promote androgen secretion via three pathways, which ultimately results in hyperandrogenemia: direct activation of 17-β hydroxylase in follicular membrane cells, which promotes androgen production; stimulation of luteinizing hormone production via insulin receptors in the pituitary gland, which further enhances androgen production in the ovaries; inhibition of the synthesis of hepatic sex hormone-binding globulins, which leads to an increase in free androgen levels in the bloodstream [62, 63]. Therefore, antiandrogens, such as oral contraceptives, play an important role in symptomatic management while treating PCOS.

BL is a new Chinese medicine prepared from *C. sinensis* mushroom powder, which has been confirmed to exert anti-PCOS effects in clinical studies [20–25] However, owing to the multicomponent and multitarget characteristics of Chinese medicinal preparations, the specific mechanisms require in-depth investigation. Therefore, to examine the molecular mechanism of BL therapy for PCOS, this study relied on network pharmacology and bioinformatics.

In this study, PCOS models were established via the subcutaneous injection of DHEA into mice combined with a high-sugar and high-fat diet. The research findings indicated that the model mice exhibited disrupted estrous cycles and evident polycystic ovary lesions, which suggested that PCOS models were successfully created. The mice were subsequently treated with BL, after which their estrous cycles and ovarian lesions tended to normalize. Additionally, when their serum testosterone and estradiol levels were tested, significant improvements were noted in the BL group. These experimental data confirmed the effectiveness of BL in DHEA-induced PCOS model mice. Nonetheless, the mechanisms underlying the ability of BL to alleviate PCOS remained unclear.

Network pharmacology, which is based on the “drug–target–gene–disease” interaction network, systematically examines the intervention and influence of drugs on the disease network. This study can reveal the material basis and effect mechanism of the synergistic effect of Chinese medicine on the organism holistically and systematically. Molecular docking is a multidisciplinary technique that combines computer technology, molecular biology, and molecular pharmacology to aid in the molecular design of drugs. Furthermore, this technique is useful in elucidating the mechanism of action of the effector components and targets of traditional Chinese medicine at the molecular level. Therefore, this research made use of network pharmacology and molecular docking to explore the potential mechanism and material basis of BL in treating PCOS.

Seven effective components were retrieved from the TCMSP database (OB ≥ 30%, DL ≥ 0.18). Of these, beta-sitosterol inhibits cholesterol absorption in the small intestine and lowers its plasma level [64], whereas arachidonic acid acts as a precursor for prostaglandin, thromboxane, and leukotriene synthesis. Furthermore, by activating the IRS-1/Akt pathway, β-Sitosterol may promote insulin release [65]. T Cerevisterol inhibits MAPK/NF-κB/AP-1 and activates the Nrf2/HO-1 signaling cascade to alleviate inflammation [66]. Linoleic acetate, peroxyergosterol, cholesterol palmitate, and cholesterol exhibit anti-inflammatory and antioxidant properties that protect blood vessels and reduce damage. The synergistic effects of these multicomponent compounds on anti-inflammation and IR regulation may serve as the material basis of BL in treating PCOS.

The GEO database is a valuable repository of high-throughput gene expression data submitted by research institutions globally. Via computer language mining and analysis of clinical data samples, this database enables rapid and comprehensive investigation of disease-related targets. Relevant differential genes can be obtained by inputting the disease name and applying conditional filtering. In this study, PCOS was searched in the GEO database and four microarray datasets (GSE1615, GSE5090, GSE5850, and GSE48301) were screened. These datasets were analyzed using the R language, which resulted in the identification of 491 differentially expressed genes (DEGs), including 130 upregulated genes and 361 downregulated genes. Additionally, disease target databases, such as GeneCards, DrugBank, OMIM, and TTD, were integrated to predict targets and establish a comprehensive database of differential genes associated with PCOS.

Network pharmacology analysis revealed that the seven active ingredients of BL corresponded to 437 predicted targets, 143 pharmacophoric cross targets, and 30 key pathways. By combining protein–protein interaction network topology analysis with related literature reports, this study predicted that ESR1, MAPK1, PPARG, CD4, PIK3CA, JAK2, MAPK8, IL2, and TLR4 were the key targets of BL in treating PCOS. PPARG is a nuclear receptor that binds to peroxisome proliferators, such as fatty acids and lipid-lowering drugs. PPARG binds to certain PPAR response elements on the DNA and, once activated by ligands, controls the transcription of its target genes, including acyl-coenzyme A oxidase. This modulation of the peroxisomal β-oxidation pathway of fatty acids is a key regulator of adipocyte development and glucose homeostasis. PPARG further inhibits NF-κB-mediated proinflammatory responses, which makes it a key regulator of intestinal homeostasis [67–69]. ESR1, a nuclear hormone receptor, and NF-κB inhibit each other in a cell-specific manner, which reduces NF-κB DNA binding activity and displaces RELA/p65 and related coregulators from the promoter. This inhibition results in reduced NF-κB-mediated transcription from the IL-6 promoter. ESR1 and NF-κB can work synergistically to induce transcription by recruiting the appropriate neighboring response elements for membrane-initiated estrogenic signaling that involves various kinase cascades [70–75].

PCOS is a chronic low-grade inflammatory disease often associated with disturbed sex hormone levels and IR. Ovarian tissues from patients have been found to contain elevated levels of IL-6 and TNF-α, which may be a major factor in the ability of PCOS to sustain low-grade inflammation. TNF-α is a pertinent inflammatory factor released by adipocytes and macrophages. Studies have shown that the upregulation of TNF-α expression induces apoptosis of follicular granulosa cells, activates mitosis of follicular membrane mesenchymal cells, decreases follicular granulosa cells, and increases follicular membrane mesenchymal cells. These alterations ultimately impair estrogen synthesis and increase androgen synthesis. TNF-α is linked to IR, ovarian follicular membrane cell growth, glucolipid metabolism control, ovarian follicular membrane cell proliferation, and steroidogenesis [76, 77]. When stimulated by receptor tyrosine kinase ligands, such as EGF, insulin, IGF1, VEGFA, and PDGF, PIK3CA plays a vital role in recruiting PH domain-containing proteins to the membrane and activating them [78, 79]. AKT1 has been extensively studied for its crucial role in islet β-cell proliferation and is expressed in oocytes, primordial follicular membrane cells, granulosa cells, and luteal cells [80]. Both innate and adaptive immunity depend on JAK2 to mediate key signaling processes. Growth hormone, prolactin, leptin, erythropoietin, thrombopoietin, or type II receptors, such as IFN-α, IFN-β, IFN-γ, and other interleukins, are its partners in the cytoplasm [81]. In summary, BL may suppress the inflammatory response in PCOS, eliminate IR, regulate sex hormone levels, and alleviate immune disorders.

Pathway enrichment analysis signified that the effects of BL in treating PCOS may be related to PI3K-Akt signaling, IR, and toll-like receptor (TLR) signaling pathways. The PI3K-Akt signaling pathway is involved in cell survival, proliferation, growth, and metabolism, among other cellular processes. This pathway is activated by the binding of ligands, such as insulin and growth factors, to their corresponding receptors on the cell membrane. This binding activates PI3K, which mediates downstream AKT, thereby affecting NF-κB and further leading to the release of inflammatory factors. TLR1-TLR10 [82], which are widely expressed in cells of the innate and adaptive immune systems, are the main receptors in humans. According to a recent study, endogenous cytokines generated from tissue damage and endogenous chemicals released from saturated fatty acids may both activate TLRs [83]. Thus, TLRs also mediate inflammation caused by endogenous molecules in addition to infection-induced inflammation. Furthermore, these receptors play a vital role in POCS inflammation [83]. A key pathological manifestation of PCOS is IR, which is linked to defective insulin activity and secretion. An investigation has alluded that IR contributes to the metabolic and reproductive pathophysiological mechanisms of the syndrome. Hyperinsulinemia is commonly associated with hyperandrogenemia in women with hyperandrogenic disorders [84]. An important pathological manifestation of PCOS is insulin resistance (IR), which is associated with defective insulin activity and secretion. Current evidence suggests that insulin resistance contributes to the metabolic and reproductive pathophysiological mechanisms of the syndrome, that hyperinsulinemia is commonly associated with hyperandrogenemia in women with hyperandrogenic disorders [85]. The presence of insulin receptors in the stroma and follicular compartment of the human ovary suggests that it is another important target organ for insulin action [86]. In addition, the pathways predicted to be affected by BL, such as endocrine resistance and thyroid hormone signaling pathways, can affect insulin activity [87, 88]. These findings suggest that BL can exert synergistic effects via multiple components, targets, and pathways.

The results of molecular docking indicated that the active ingredients of BL have a high binding affinity for their targets, particularly JAK2-cholesteryl palmitate, PIK3CA–cholesteryl palmitate, PPARG–arachidonic acid, and PPARG–linoleyl acetate, which exhibited total scores of 10.719, 10.453, 10.321, and 9.613, respectively. These results imply that PI3K-Akt, TLR signaling, and IR pathways may be important for the efficacy of BL. These pathways may also play a crucial role in the effectiveness of BL. However, further investigations are required to confirm these results and determine the potential therapeutic applications of BL for managing diseases related to these pathways.

Via analysis of the GEO database, samples were selected from an obese PCOS model that was compatible with the mouse model created using a high-sugar and high-fat diet and subcutaneous injection of DHEA. ANOVA was performed using R 4.2.3 with standard selection criteria of |logFC|≥ 1 and adjusted *p*-value < 0.05 screening to obtain DEGs. The analysis revealed the upregulation of core targets *JAK2*, *PPARG*, *PI3K*, and *AKT1* as well as downregulation of *ESR1* and *IRS1*.

Overactivation of PI3K-Akt signaling has been reported in certain studies in patients with PCOS [89, 90], but downregulation of this signaling has also been observed under different study conditions [91]. This difference could be attributed to ethnic variations and study conditions. qPCR analysis of gene expression in ovarian tissues of PCOS model mice confirmed these findings, which agreed with the results of bioinformatics-based prediction analysis in human PCOS. Additionally, a substantial difference in gene expression was noted between the BL treatment group and the model group, suggesting that BL may exert anti-PCOS effects by modulating the PI3K-Akt pathway, the TLR signaling system, and the IR route. However, further studies are needed to validate these findings and determine the clinical implications of using BL to manage PCOS.

## Conclusion

This study made use of bioinformatics, network pharmacology, molecular docking, and genetic validation to identify the active components, probable target pathways, and molecular processes of BL in preventing and treating PCOS. Linoleyl acetate, cholesteryl palmitate, and arachidonic acid were identified as the main components of BL in treating PCOS. These compounds affected the JAK-STAT and PI3K-Akt signaling pathways by binding to JAK2, PIK3CA, PPARG, ESR1, IRS1, and AKT. They improved IR and inflammatory response and exhibited multitarget and multi-pathway therapeutic effects in anti-PCOS. These findings could serve as a solid foundation for additional investigations on the effectiveness and molecular processes of BL in treating PCOS.

## Data Availability

All data generated or analysed during this study are included in this published article.

## Abbreviations

PCOS: Polycystic ovary syndrome
TCMSP: Traditional Chinese Medicine Systems Pharmacology Database and Analysis Platform
GEO: Gene expression omnibus
BL: Bailing Capsule
DHEA: Dehydrogenated epiandrosterone
PPI: Protein-Protein Interaction
BP: Biological processes
CC: Cell components
MF: Molecular functions
DEGs: Differentially expressed genes
T: Testosterone
E2: Estradiol
PI3K: Phosphoinositide 3-kinase
AKT: Protein kinase B
JAK2: Tyrosine-protein kinase JAK2
PPARG: Peroxisome proliferator-activated receptor gamma
ESR1: Estrogen receptor
SRC: Proto-oncogene tyrosine-protein kinase Src
IRS1: Insulin receptor

## References

[1] Escobar-Morreale HF (2018). Polycystic ovary syndrome: definition, aetiology, diagnosis and treatment. Nat Rev Endocrinol.

[2] Walter K (2022). What is polycystic ovary syndrome?. JAMA.

[3] Cooney LG, Dokras A (2018). Beyond fertility: polycystic ovary syndrome and long-term health. Fertil Steril.

[4] Patel S (2018). Polycystic ovary syndrome (PCOS), an inflammatory, systemic, lifestyle endocrinopathy. J Steroid Biochem Mol Biol.

[5] Huddleston HG, Dokras A (2022). Diagnosis and treatment of polycystic ovary syndrome. JAMA.

[6] Yildiz BO, Bozdag G, Yapici Z, Esinler I, Yarali H (2012). Prevalence, phenotype and cardiometabolic risk of polycystic ovary syndrome under different diagnostic criteria. Hum Reprod.

[7] Legro RS, Arslanian SA, Ehrmann DA, Hoeger KM, Murad MH, Pasquali R, Welt CK (2013). Diagnosis and treatment of polycystic ovary syndrome: an Endocrine Society clinical practice guideline. J Clin Endocrinol Metab.

[8] Domecq JP, Prutsky G, Mullan RJ, Sundaresh V, Wang AT, Erwin PJ, Welt C, Ehrmann D, Montori VM, Murad MH (2013). Adverse effects of the common treatments for polycystic ovary syndrome: a systematic review and meta-analysis. J Clin Endocrinol Metab.

[9] Legro RS, Barnhart HX, Schlaff WD, Carr BR, Diamond MP, Carson SA, Steinkampf MP, Coutifaris C, McGovern PG, Cataldo NA (2007). Clomiphene, metformin, or both for infertility in the polycystic ovary syndrome. N Engl J Med.

[10] Morley LC, Tang T, Yasmin E, Norman RJ, Balen AH (2017). Insulin-sensitising drugs (metformin, rosiglitazone, pioglitazone, D-chiro-inositol) for women with polycystic ovary syndrome, oligo amenorrhoea and subfertility. Cochrane Database Syst Rev.

[11] Lin MJ, Chen HW, Liu PH, Cheng WJ, Kuo SL, Kao MC (2019). The prescription patterns of traditional Chinese medicine for women with polycystic ovary syndrome in Taiwan: a nationwide population-based study. Medicine (Baltimore).

[12] Liao WT, Chiang JH, Li CJ, Lee MT, Su CC, Yen HR (2018). Investigation on the use of traditional Chinese medicine for polycystic ovary syndrome in a nationwide prescription database in Taiwan. J Clin Med.

[13] Shen W, Jin B, Pan Y, Han Y, You T, Zhang Z, Qu Y, Liu S, Zhang Y (2021). The effects of traditional Chinese medicine-associated complementary and alternative medicine on women with polycystic ovary syndrome. Evid Based Complement Alternat Med.

[14] Li X, Ma J, Guo L, Dong C, Zhu G, Hong W, Chen C, Wang H, Wu X (2022). Identification of bioactive compounds and potential mechanisms of Kuntai capsule in the treatment of polycystic ovary syndrome by integrating network pharmacology and bioinformatics. Oxid Med Cell Longev.

[15] Ma K (2021). Advantages of integrated Chinese and western medicine in diagnosis and treatment of anovulatory infertility due to kidney deficiency and blood stasis. Zhongguo Zhong Yao Za Zhi.

[16] Balen AH, Morley LC, Misso M, Franks S, Legro RS, Wijeyaratne CN, Stener-Victorin E, Fauser BC, Norman RJ, Teede H (2016). The management of anovulatory infertility in women with polycystic ovary syndrome: an analysis of the evidence to support the development of global WHO guidance. Hum Reprod Update.

[17] Jin P, Xie Y (2018). Treatment strategies for women with polycystic ovary syndrome. Gynecol Endocrinol.

[18] Huang BM, Hsiao KY, Chuang PC, Wu MH, Pan HA, Tsai SJ (2004). Upregulation of steroidogenic enzymes and ovarian 17beta-estradiol in human granulosa-lutein cells by Cordyceps sinensis mycelium. Biol Reprod.

[19] Zhang Q, Xiao X, Li M, Yu M, Ping F (2022). Bailing capsule (Cordyceps sinensis) ameliorates renal triglyceride accumulation through the PPARalpha pathway in diabetic rats. Front Pharmacol.

[20] Li YL, Ruan XY, Zhao Y, Du J, Wang LJ, Cui YM, Alfred OM (2016). Effects of Bailing capsules on metabolism in patients with polycystic ovary syndrome. J Cap Med Univ.

[21] Li XH, Xue X, Ha LX, Liu CL, Chen Q, Lu XN (2017). Effects of Bailing capsule on the folicular fluid bone morphogenetic protein, growth differentiation factor-9 and insulin like growth factor of patients with polycystic ovary syndrome. Prog Mod Biomed.

[22] Feng HF, Du QM, Huang CP (2018). Bailing capsules combined with metformin has effect on metabolism indexes and sex hormone of polycystic ovary syndrome. New Chin Med.

[23] Liu N, Pi D, Liu CM (2018). Effect of Balling capsule combined with ethinylestradiol and cyproterone acetate and metformin on lipid metabolism and insulin resistance in patients with polycystic ovary syndrome. J Hainan Med Univ.

[24] Zhang YC, Li LL, Shi HX, Ren W (2018). Effect of Bailing capsule combined with Ietrozole on endometrial thickness and serum levels of lGF-1 and visfatin in patients with polycystic ovany syndrome. J Guangxi Med Univ.

[25] Zhu JY, Liu J, Cao XJ, Wang XY (2021). An efficacy and feasibility analysis of Chinese patent medicine combined with letrozole in the treatment of women with ovulation disorders: a network meta-analysis. Front Pharmacol.

[26] Sun W, Qing R, Fan Z, He Q, Wu J, He Y, Ouyang L, Chen Z, Deng G (2023). Mechanism of Wuyao-ginseng medicine pair in the prevention and treatment of diarrhea-type irritable bowel syndrome based on gene expression omnibus chip data. Life (Basel).

[27] Wu Z, Pan X, Deng C, Cai M, Yuan K, Huang P, Shi G (2022). Mechanism of herb pairs Astragalus mongholicus and Curcuma phaeocaulis valeton in treating gastric carcinoma: a network pharmacology combines with differential analysis and molecular docking. Evid Based Complement Alternat Med.

[28] Poojary PS, Nayak G, Panchanan G, Rao A, Kundapur SD, Kalthur SG, Mutalik S, Adiga SK, Zhao Y, Bakkum-Gamez J (2022). Distinctions in PCOS induced by letrozole vs dehydroepiandrosterone with high-fat diet in mouse model. Endocrinology.

[29] Ru J, Li P, Wang J, Zhou W, Li B, Huang C, Li P, Guo Z, Tao W, Yang Y (2014). TCMSP: a database of systems pharmacology for drug discovery from herbal medicines. J Cheminform.

[30] Xu X, Zhang W, Huang C, Li Y, Yu H, Wang Y, Duan J, Ling Y (2012). A novel chemometric method for the prediction of human oral bioavailability. Int J Mol Sci.

[31] Zhang YF, Huang Y, Ni YH, Xu ZM (2019). Systematic elucidation of the mechanism of geraniol via network pharmacology. Drug Des Devel Ther.

[32] Tao W, Xu X, Wang X, Li B, Wang Y, Li Y, Yang L (2013). Network pharmacology-based prediction of the active ingredients and potential targets of Chinese herbal Radix Curcumae formula for application to cardiovascular disease. J Ethnopharmacol.

[33] Ban C, Jo M, Park YH, Kim JH, Han JY, Lee KW, Kweon DH, Choi YJ (2020). Enhancing the oral bioavailability of curcumin using solid lipid nanoparticles. Food Chem.

[34] Daina A, Michielin O, Zoete V (2019). SwissTargetPrediction: updated data and new features for efficient prediction of protein targets of small molecules. Nucleic Acids Res.

[35] Keiser MJ, Roth BL, Armbruster BN, Ernsberger P, Irwin JJ, Shoichet BK (2007). Relating protein pharmacology by ligand chemistry. Nat Biotechnol.

[36] UniProt CT (2018). UniProt: the universal protein knowledgebase. Nucleic Acids Res.

[37] Wang Y, Yuan Y, Wang W, He Y, Zhong H, Zhou X, Chen Y, Cai XJ, Liu LQ (2022). Mechanisms underlying the therapeutic effects of Qingfeiyin in treating acute lung injury based on GEO datasets, network pharmacology and molecular docking. Comput Biol Med.

[38] Ritchie ME, Phipson B, Wu D, Hu Y, Law CW, Shi W, Smyth GK (2015). limma powers differential expression analyses for RNA-sequencing and microarray studies. Nucleic Acids Res.

[39] Steenwyk JL, Rokas A (2021). ggpubfigs: colorblind-friendly color palettes and ggplot2 graphic system extensions for publication-quality scientific figures. Microbiol Resour Announc.

[40] Rebhan M, Chalifa-Caspi V, Prilusky J, Lancet D (1997). GeneCards: integrating information about genes, proteins and diseases. Trends Genet.

[41] Wishart DS, Knox C, Guo AC, Shrivastava S, Hassanali M, Stothard P, Chang Z, Woolsey J (2006). DrugBank: a comprehensive resource for in silico drug discovery and exploration. Nucleic Acids Res.

[42] Amberger JS, Bocchini CA, Schiettecatte F, Scott AF, Hamosh A (2015). OMIM.org: Online Mendelian Inheritance in Man (OMIM(R)), an online catalog of human genes and genetic disorders. Nucleic Acids Res.

[43] Wang Y, Zhang S, Li F, Zhou Y, Zhang Y, Wang Z, Zhang R, Zhu J, Ren Y, Tan Y (2020). Therapeutic target database 2020: enriched resource for facilitating research and early development of targeted therapeutics. Nucleic Acids Res.

[44] Chen C, Chen H, Zhang Y, Thomas HR, Frank MH, He Y, Xia R (2020). TBtools: an integrative toolkit developed for interactive analyses of big biological data. Mol Plant.

[45] Szklarczyk D, Gable AL, Lyon D, Junge A, Wyder S, Huerta-Cepas J, Simonovic M, Doncheva NT, Morris JH, Bork P (2019). STRING v11: protein-protein association networks with increased coverage, supporting functional discovery in genome-wide experimental datasets. Nucleic Acids Res.

[46] Doncheva NT, Morris JH, Gorodkin J, Jensen LJ (2019). Cytoscape StringApp: network analysis and visualization of proteomics data. J Proteome Res.

[47] Chin CH, Chen SH, Wu HH, Ho CW, Ko MT, Lin CY (2014). cytoHubba: identifying hub objects and sub-networks from complex interactome. BMC Syst Biol.

[48] Bader GD, Hogue CW (2003). An automated method for finding molecular complexes in large protein interaction networks. BMC Bioinformatics.

[49] Zhou Y, Zhou B, Pache L, Chang M, Khodabakhshi AH, Tanaseichuk O, Benner C, Chanda SK (2019). Metascape provides a biologist-oriented resource for the analysis of systems-level datasets. Nat Commun.

[50] Kanehisa M, Goto S (2000). KEGG: kyoto encyclopedia of genes and genomes. Nucleic Acids Res.

[51] Kanehisa M (2019). Toward understanding the origin and evolution of cellular organisms. Protein Sci.

[52] Kanehisa M, Furumichi M, Sato Y, Kawashima M, Ishiguro-Watanabe M (2023). KEGG for taxonomy-based analysis of pathways and genomes. Nucleic Acids Res.

[53] Huang DW, Sherman BT, Lempicki RA (2009). Systematic and integrative analysis of large gene lists using DAVID bioinformatics resources. Nat Protoc.

[54] Tang D, Chen M, Huang X, Zhang G, Zeng L, Zhang G, Wu S, Wang Y (2023). SRplot: a free online platform for data visualization and graphing. PLoS One.

[55] Wang R, Lu Y, Wang S (2003). Comparative evaluation of 11 scoring functions for molecular docking. J Med Chem.

[56] Joham AE, Teede HJ, Ranasinha S, Zoungas S, Boyle J (2015). Prevalence of infertility and use of fertility treatment in women with polycystic ovary syndrome: data from a large community-based cohort study. J Womens Health (Larchmt).

[57] Wang J, Wu D, Guo H, Li M (2019). Hyperandrogenemia and insulin resistance: the chief culprit of polycystic ovary syndrome. Life Sci.

[58] Chen Y, Wang XJ, Jin HL, Jin L (2013). Effects of resolving method of Chinese medicine on the lipid metabolism in polycystic ovary syndrome accompanied with non-alcoholic fatty liver disease. Zhongguo Zhong Xi Yi Jie He Za Zhi.

[59] Jeanes YM, Reeves S (2017). Metabolic consequences of obesity and insulin resistance in polycystic ovary syndrome: diagnostic and methodological challenges. Nutr Res Rev.

[60] Xu Y, Wu Y, Huang Q (2017). Comparison of the effect between pioglitazone and metformin in treating patients with PCOS: a meta-analysis. Arch Gynecol Obstet.

[61] Kupreeva M, Diane A, Lehner R, Watts R, Ghosh M, Proctor S, Vine D (2019). Effect of metformin and flutamide on insulin, lipogenic and androgen-estrogen signaling, and cardiometabolic risk in a PCOS-prone metabolic syndrome rodent model. Am J Physiol Endocrinol Metab.

[62] Mellati AA, Sharifi F, Sajadinejad M, Sohrabi D, Mazloomzadeh S (2012). The relationship between retinol-binding protein 4 levels, insulin resistance, androgen hormones and polycystic ovary syndrome. Scand J Clin Lab Invest.

[63] Ye W, Xie T, Song Y, Zhou L (2021). The role of androgen and its related signals in PCOS. J Cell Mol Med.

[64] Bin SM, Karim S, Sharmin T, Morshed MM (2016). Critical analysis on characterization, systemic effect, and therapeutic potential of beta-sitosterol: a plant-derived orphan phytosterol. Medicines (Basel).

[65] Babu S, Krishnan M, Rajagopal P, Periyasamy V, Veeraraghavan V, Govindan R, Jayaraman S (2020). Beta-sitosterol attenuates insulin resistance in adipose tissue via IRS-1/Akt mediated insulin signaling in high fat diet and sucrose induced type-2 diabetic rats. Eur J Pharmacol.

[66] Alam MB, Chowdhury NS, Sohrab MH, Rana MS, Hasan CM, Lee SH (2020). Cerevisterol alleviates inflammation via suppression of MAPK/NF-kappaB/AP-1 and activation of the Nrf2/HO-1 signaling cascade. Biomolecules.

[67] Yin Y, Yuan H, Wang C, Pattabiraman N, Rao M, Pestell RG, Glazer RI (2006). 3-phosphoinositide-dependent protein kinase-1 activates the peroxisome proliferator-activated receptor-gamma and promotes adipocyte differentiation. Mol Endocrinol.

[68] Park SH, Choi HJ, Yang H, Do KH, Kim J, Lee DW, Moon Y (2010). Endoplasmic reticulum stress-activated C/EBP homologous protein enhances nuclear factor-kappaB signals via repression of peroxisome proliferator-activated receptor gamma. J Biol Chem.

[69] Mukherjee R, Jow L, Croston GE, Paterniti JJ (1997). Identification, characterization, and tissue distribution of human peroxisome proliferator-activated receptor (PPAR) isoforms PPARgamma2 versus PPARgamma1 and activation with retinoid X receptor agonists and antagonists. J Biol Chem.

[70] Porter W, Saville B, Hoivik D, Safe S (1997). Functional synergy between the transcription factor Sp1 and the estrogen receptor. Mol Endocrinol.

[71] Stein B, Yang MX (1995). Repression of the interleukin-6 promoter by estrogen receptor is mediated by NF-kappa B and C/EBP beta. Mol Cell Biol.

[72] Pradhan M, Bembinster LA, Baumgarten SC, Frasor J (2010). Proinflammatory cytokines enhance estrogen-dependent expression of the multidrug transporter gene ABCG2 through estrogen receptor and NFkappaB cooperativity at adjacent response elements. J Biol Chem.

[73] Gionet N, Jansson D, Mader S, Pratt MA (2009). NF-kappaB and estrogen receptor alpha interactions: Differential function in estrogen receptor-negative and -positive hormone-independent breast cancer cells. J Cell Biochem.

[74] Nettles KW, Gil G, Nowak J, Metivier R, Sharma VB, Greene GL (2008). CBP Is a dosage-dependent regulator of nuclear factor-kappaB suppression by the estrogen receptor. Mol Endocrinol.

[75] Liu H, Liu K, Bodenner DL (2005). Estrogen receptor inhibits interleukin-6 gene expression by disruption of nuclear factor kappaB transactivation. Cytokine.

[76] Yu Y, Li G, He X, Lin Y, Chen Z, Lin X, Xu H (2021). MicroRNA-21 regulate the cell apoptosis and cell proliferation of polycystic ovary syndrome (PCOS) granulosa cells through target toll like receptor TLR8. Bioengineered.

[77] Mohammadi S, Kayedpoor P, Karimzadeh-Bardei L, Nabiuni M (2017). The effect of curcumin on TNF-alpha, IL-6 and CRP expression in a model of polycystic ovary syndrome as an inflammation state. J Reprod Infertil.

[78] Easton JB, Kurmasheva RT, Houghton PJ (2006). IRS-1: auditing the effectiveness of mTOR inhibitors. Cancer Cell.

[79] Leroy C, Ramos P, Cornille K, Bonenfant D, Fritsch C, Voshol H, Bentires-Alj M (2016). Activation of IGF1R/p110beta/AKT/mTOR confers resistance to alpha-specific PI3K inhibition. Breast Cancer Res.

[80] Zhao H, Zhou D, Chen Y, Liu D, Chu S, Zhang S (2017). Beneficial effects of Heqi san on rat model of polycystic ovary syndrome through the PI3K/AKT pathway. Daru.

[81] Sakatsume M, Igarashi K, Winestock KD, Garotta G, Larner AC, Finbloom DS (1995). The Jak kinases differentially associate with the alpha and beta (accessory factor) chains of the interferon gamma receptor to form a functional receptor unit capable of activating STAT transcription factors. J Biol Chem.

[82] Fitzgerald KA, Kagan JC (2020). Toll-like receptors and the control of immunity. Cell.

[83] Brunn GJ, Bungum MK, Johnson GB, Platt JL (2005). Conditional signaling by Toll-like receptor 4. FASEB J.

[84] Wang Y, He J, Yang J (2018). Eicosapentaenoic acid improves polycystic ovary syndrome in rats via sterol regulatory element-binding protein 1 (SREBP-1)/toll-like receptor 4 (TLR4) pathway. Med Sci Monit.

[85] Diamanti-Kandarakis E, Papavassiliou AG (2006). Molecular mechanisms of insulin resistance in polycystic ovary syndrome. Trends Mol Med.

[86] Poretsky L, Smith D, Seibel M, Pazianos A, Moses AC, Flier JS (1984). Specific insulin binding sites in human ovary. J Clin Endocrinol Metab.

[87] Guo XD, Zhang DY, Gao XJ, Parry J, Liu K, Liu BL, Wang M (2013). Quercetin and quercetin-3-O-glucuronide are equally effective in ameliorating endothelial insulin resistance through inhibition of reactive oxygen species-associated inflammation. Mol Nutr Food Res.

[88] Luo C, Yang H, Tang C, Yao G, Kong L, He H, Zhou Y (2015). Kaempferol alleviates insulin resistance via hepatic IKK/NF-kappaB signal in type 2 diabetic rats. Int Immunopharmacol.

[89] Villavicencio A, Goyeneche A, Telleria C, Bacallao K, Gabler F, Fuentes A, Vega M (2009). Involvement of Akt, Ras and cell cycle regulators in the potential development of endometrial hyperplasia in women with polycystic ovarian syndrome. Gynecol Oncol.

[90] Nekoonam S, Naji M, Nashtaei MS, Mortezaee K, Koruji M, Safdarian L, Amidi F (2017). Expression of AKT1 along with AKT2 in granulosa-lutein cells of hyperandrogenic PCOS patients. Arch Gynecol Obstet.

[91] Gong Y, Luo S, Fan P, Zhu H, Li Y, Huang W (2020). Growth hormone activates PI3K/Akt signaling and inhibits ROS accumulation and apoptosis in granulosa cells of patients with polycystic ovary syndrome. Reprod Biol Endocrinol.

